# Sawmill waste derived carbon dots as a fluorescent probe for synthetic dyes in soft drinks

**DOI:** 10.1038/s41598-021-97552-5

**Published:** 2021-09-09

**Authors:** Datta B. Gunjal, Laxman S. Walekar, Samadhan P. Pawar, Prashant V. Anbhule, Mukund G. Mali, Vinayak P. Dhulap, Daewon Sohn, Prasad G. Mahajan, Ki Hwan Lee, Rajendra V. Shejwal, Govind B. Kolekar

**Affiliations:** 1 Fluorescence Spectroscopy Research Laboratory, Department of Chemistry, Shivaji University, Kolhapur, Maharashtra 416004 India; 2 Department of Chemistry, Lal Bahadur Shastri College of Arts, Science and Commerce, Satara, Maharashtra 415002 India; 3 School of Chemical Sciences, Punyashlok Ahilyadevi Holkar, Solapur University, Solapur, Maharashtra 413255 India; 4 School of Earth Sciences, Punyashlok Ahilyadevi Holkar, Solapur University, Solapur, Maharashtra 413255 India; 5 Department of Chemistry and the Research Institute for Convergence of Basic Science, Hanyang University, Seoul, 04763 Republic of Korea; 6 Vidya Prathisthan’s Arts, Commerce and Science College, Vidyanagari, Baramati, Maharashtra 413133 India; 7 Department of Chemistry, Kongju National University, Gongju, Chungnam 32588 Republic of Korea

**Keywords:** Chemistry, Materials science, Nanoscience and technology

## Abstract

Herein, for the first time the carbon dots (CDs) were synthesized by reflux method from sawmill waste material. We also represent a novel strategy based on fluorescent CDs for determination of ponceau 4R and allura red dyes in soft drinks. Interestingly, both the dyes were sensitive and showed effective fluorescence quenching of the CDs owing to the interaction between them. The analytical applicability of CDs were evaluated for detection of both the dyes with a good linear relationship between the concentration range of 0.0 to 3.0 µg mL^−1^ and having detection limit 0.45 and 0.47 µg mL^−1^ for allura red and ponceau 4R dyes respectively. Meanwhile, the potential application of this novel fluorescent probe for dyes determination in real samples was validated in different soft drink samples with good accuracy and precision. Thus, these findings provides new insights for the potential risk assessment of both the dyes. Moreover, CDs acted as an excellent fluorescent material in cellular imaging owing to their cellular uptake and localization.

## Introduction

Decent health has utmost importance throughout the life of human beings. It depends on so many factors such as exercise, proper nutrients, stress management and most vital healthy food. Among these healthy or organic food have attracted numerous attention in recent years owing to their tremendous benefits. But, recently food safety became major concern of the people because of contamination and excessive additives in food. Food additives have been employed to improve the appearance, freshness, nutritional value, texture and taste of food. Synthetic dyes or colorant is commonly used additives in foodstuffs because of their intriguing properties such as brightness, high stability, more cost-efficient and excellent color uniformity and ultimately food becomes appealing. These colorants are acceptable up to the certain extent, described by food regulation bodies. However, excessive consumption of these dyes poses some potential risks such as carcinogenic, mutagenic and teratogenic effect to human health^1^.

Among the different synthetic dyes Allura Red and Ponceau-4R, as water-soluble, red colored, synthetic azo dyes, are widely used in various foods such as drinks, jelly, fruit juice, candies, sweets and other foods in India. Despite of the overwhelming applications of these synthetic dyes, their toxic effect could not be ignored. The overconsumption of azo dyes leading hyperactivity, physical and mental affect in children, develop insomnia, asthma and allergies, frequent headaches in adults^2,3^. Therefore, their amount into food is strictly controlled by laws and regulations at national as well as international level to ensure the consumers safety. European Food Safety Authority (EFSA) have set permissible concentrations of Allura Red is 0–7 mg/kg/bw/day while acceptable daily intake for Ponceau 4R is 0.025–0.5 g/kg (GB2760-2011) depending on the type of food. Thus, quality of food and eventually safety of consumer is inevitably depending on the additives of food. So, foodstuffs having synthetic dyes must pass a thorough quality control and development of simple, rapid, convenient analytical method for the simultaneous determination of Ponceau-4R and Allura Red in foodstuffs is of great importance.

So far different analytical techniques have been adapted for the determination of Ponceau-4R [PR] and Allura Red [AR], such as spectrophotometry^1,4^ high-performance liquid chromatography^5,6^, differential pulse polarography^7^, LC–MS method^8^ and electrochemical methods^9,10^. However, these methods are expensive, time consuming, complicated to operate, and requires extensive sample preparations. While electrochemical methods have less sensitivity and stability which confronts precise use of this method. Therefore, to develop rapid, simple and sensitive analytical method for the quantification PR and AR is still of challenge. Among the different analysis techniques, fluorescence spectroscopy has been widely employed method since the years owing to their flexible, sensitive, rapid, non-destructive characteristics. Therefore, considerable efforts have been made to explore efficient fluorescent probe for detection of various ions, biomolecules from the past several years^11,12^. Nevertheless, there is not a single report of analysis of PR and AR dyes by the sensitive and selective fluorescence techniques till date. This observation reveals much scope in analysis of dyes with fluorescence spectroscopy.

The objective of this work is to develop low cost, rapid and sensitive fluorescent probe and eventually a method for the quantification of PR and AR dyes. So, recently discovered carbon dots (CDs) is right choice to make method valuable and simple. This carbon family nanomaterial discovered in 2004 and garnered much attention owing to their outstanding properties, such as excellent aqueous solubility, biocompatibility, chemical inertness, low photobleaching, easy fabrication and less toxicity^13^. In line for these merits, scientists have paid attention on their synthesis and practical applications in various fields. Up-to-date, different techniques have been employed for their synthesis, laser ablation, electrochemical oxidation of graphite, microwave assisted, hydrothermal, ultrasonication and chemical oxidation^14^. Moreover, CDs were applied in numerous fields such as in sensor^15^, photocatalyst^16^, bioimaging^17^ drug delivery^18^ and optoelectonics^19–22^. However, CDs are rarely employed for assay of PR and AR. Therefore, the detection of PR and AR based on the fluorescence quenching of CDs is still innovative and meaningful.

In this work, we have synthesize CDs from copious sawmill waste material by chemical oxidation method. This low-cost CDs based method was further validated as highly sensitive and reliable fluorescent probe for the quantification of PR and AR with a satisfactory analytical performance. More importantly, their analytical application is demonstrated in real samples. Moreover, these CDs exhibited excellent multicolour imaging function in the living cells.

## Materials and methods

### Materials

The standard Ponceau-4R and Allura Red dyes were brought from TCI chemicals (India) pvt. Ltd. Citric acid, sucrose, Boric acid, tri-sodium phosphate dodecahydrate, glucose, sodium citrate, sodium chloride and potassium chloride were purchased from sigma-aldrich (India). Soft drink samples were purchased from local market for real sample analysis. All other chemicals were of analytical grade and used without further purification, procured from sigma-aldrich (India). Double distilled water was used priory for stock solution preparation and execution of experimental work.

### Apparatus

The fluorescence spectrums were measured by an FP-8300 fluorescence spectrophotometer (Jasco, Japan) using a 1 cm path length and 5 nm slit width for both excitation and emission filter. The UV–Vis absorption spectra were acquired by a Specord Analytica Jena spectrophotometer. Transmission electron microscope (PHILIPS, CM-200 model) was used to determine the particle size and morphology with an accelerating voltage 20-200kv.The functional groups of the CDs were analysed by fourier transform infrared spectrophotometer (Jasco 680-plus Tokyo, Japan). The crystallinity of sample was explored by X-ray diffraction (XRD) spectrum, performed onBruker X-ray diffractometer (Germany) with the voltage of 45 kV and current of 40 mA. The chemical composition of sample was determined through X-ray photoelectron spectroscopy (XPS) (ThermoScientific). The fluorescence lifetime was acquired by Time Correlated Single Photon Counting (TCSPC) Spectrophotometer (HORIBA JobinYvon IBH, nano LED-07) at room temperature. Zeiss Axio-scope A 1 trinocular phase contrast fluorescent microscope was used forcell imaging at different excitation wavelength.

### Synthesis of CDs

Sawmill waste is abundantly available cellulose rich biomass hence it was exercised as source for CDs synthesis. In a typical experiment, firstly activated carbon was obtained from sawmill waste. The 0.5 g activated carbon and 50 mL HNO_3_ (1 mol/L) was mixed and sonicated for 10 min and subjected to reflux for 12 h. The colour of the solution was changed from colourless to brownish due to the formation of CDs. The resultant solution was cooled to room temperature and centrifuged at 4500 rpm for 15 min to remove larger or agglomerated carbon particles. Afterwards, supernatant was collected and neutralized with Na_2_CO_3_ followed by filtration with 0.22 µm membrane to eliminate the larger particles. Finally, pure CDs solution was obtained by dialysis through dialysis membrane (MWCO of 3500 D) against double distilled water for 24 h and stored at freeze temperature for further experiment^23^. The experimental procedure for preparation of pure CDs is graphically shown in Figure S1.

### Quantum yield measurement

Quantum yield (QY) of synthesised CDs was investigated with standard quinine sulphate solution (QY = 54%, 0.1 M H_2_SO_4_). The fluorescence intensity and absorbance values of CDs were compared with reference compound. Re-absorption effect of solution was avoided by keeping the absorbance of CDs solution below 0.10. The quantum yield was calculated by the following equation:

1 $$QY=Q{Y}_{R}\times \frac{I}{{I}_{R}}\times \frac{{A}_{R}}{A}\times \frac{{\eta }^{2}}{{\eta }_{R}^{2}}$$

where, QY is the quantum yield of CDs, I is the integrated emission intensity, A represents the absorbance while η is the refractive index of the solvents. The subscript R refers to the standard compound. The QY of CDs was calculated and found to be 3.75%.

### Detection of synthetic colorants

In a typical run, 0.5 ml diluted CDs and varying volume of standard allura red (0.05–3.0 mL) solution were placed in a 5.0 ml volumetric flask and final volume of the mixture was adjusted with distilled water. The whole system was incubated at room temperature for 10 min. Then, fluorescence spectrum of each solution was measured using a 1 cm quartz cell at 320 nm excitation wavelength while excitation and emission slit width set at 5 nm. Same experimental procedure was repeated for ponceau 4R dye. The fluorescence quenching efficiency for both the dye was calculated by plotting the F_0_/F against concentration of dyes, where F and F_0_ refer to the intensity of sample with and without analyte.

### Analysis of sample

Two different soft drink samples were collected from local market. They were boiled for 5 min to remove dissolved CO_2_ and used further without any treatment. In short, three different concentrations of standard dyes solutions were mixed with 1.0 mL sample solution and 0.5 mL CDs solutions and subjected to analysis of aforementioned procedure. The standard addition method was employed for the validation of proposed the method.

### Cell labelling assay

The saccharomyces cerevisiae, a yeast cell strain, were cultivated and grown in yeast extract-peptone-dextrose (YPD) (a mixture of glucose, yeast extract, (bacto peptone) medium at suitable temperature and with intermittent shaking. The fully-grown cell culture was aseptically withdrawn and further subjected for cell labelling assay. Briefly cell staining experiment is described as: firstly, the collected S. cerevisiae strain was washed twice by phosphate buffered saline (PBS, pH 7.4) to remove adhered unwanted material and dead cells. The cell suspension was re-suspended in PBS solution and fixed with 70% (v/v) ethanol at 4 °C for 5 min. Furthermore, for internalization of CDs, these fixed cells culture were mixed with 40 μL of carbon dot solution and incubated at room temperature for 3 h. This CDs treated cell culture was again washed twice with PBS solution and finally, a little volume of such a treated sample was mounted on glass slide for confocal fluorescence microscopic analysis. The Zeiss Axio-scope A 1 trinocular phase contrast fluorescent microscope was employed to take fluorescent images at the different excitation (408, 488, and 561 nm) wavelength.

## Result and discussion

### Characterisation

**Figure 1:**
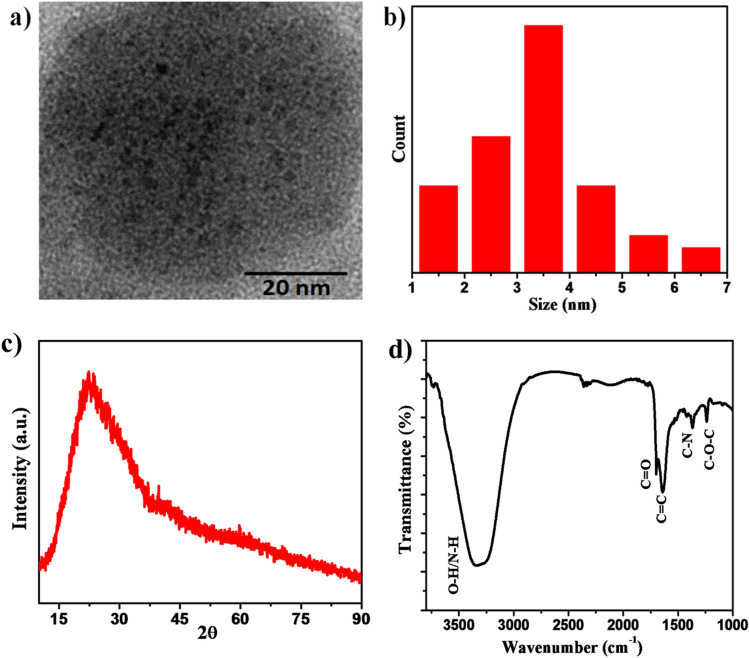
(**a**) TEM image and (**b)** particle size distribution of synthesized CDs. (**c)** XRD spectra and (**d**) FT-IR spectra of CDs.

The size and morphology of the CDs were determined by TEM analysis. Figure 1a shows the TEM image of as-synthesized CDs revealing that these carbon dots are well dispersed without any aggregation and exhibited nearly spherical shape morphology. The particle size distribution demonstrated that the spherical CDs have small size distribution ranging from 1 to 5 nm, with an average size of 3.7 nm (Fig. 1b. Moreover the average particle size of CDs also passed by the dynamic light scattering technique and it was found to be 4.75 nm which is very close to the particle size obtained by TEM (Figure S2).The XRD was employed to know the phase and degree of crystallinity of the synthesised material. The XRD pattern (Fig. 1c) of the CDs displayed broad peak centred at 23^0^ (0.82 nm) which corresponds to highly disordered and amorphous nature of carbon structure^24^.

The different surface functional groups of CDs were identified by FT-IR spectra (Fig. 1d).The characteristic absorption peak at 3339 cm^−1^ is assigned to the stretching vibrations of O–H/N–H bond. The peak around1632 cm^−1^ correspond to the C=C stretch of the carbon skeleton of CDs while shoulder like peak at 1705 adjacent to the C=C mode of vibrations was attributed to the C=O stretching. In addition, the frequency of vibrations of C–N and C–O–C bonds were exhibited by absorption peaks at 1372 and 1230 cm^−1^ respectively^25^. These peaks in IR spectrum indicated the existence of large numbers of residual carboxyl, hydroxyl groups on the surface of CDs which improves the hydrophilicity and stability of CDs in aqueous systems.

**Figure 2:**
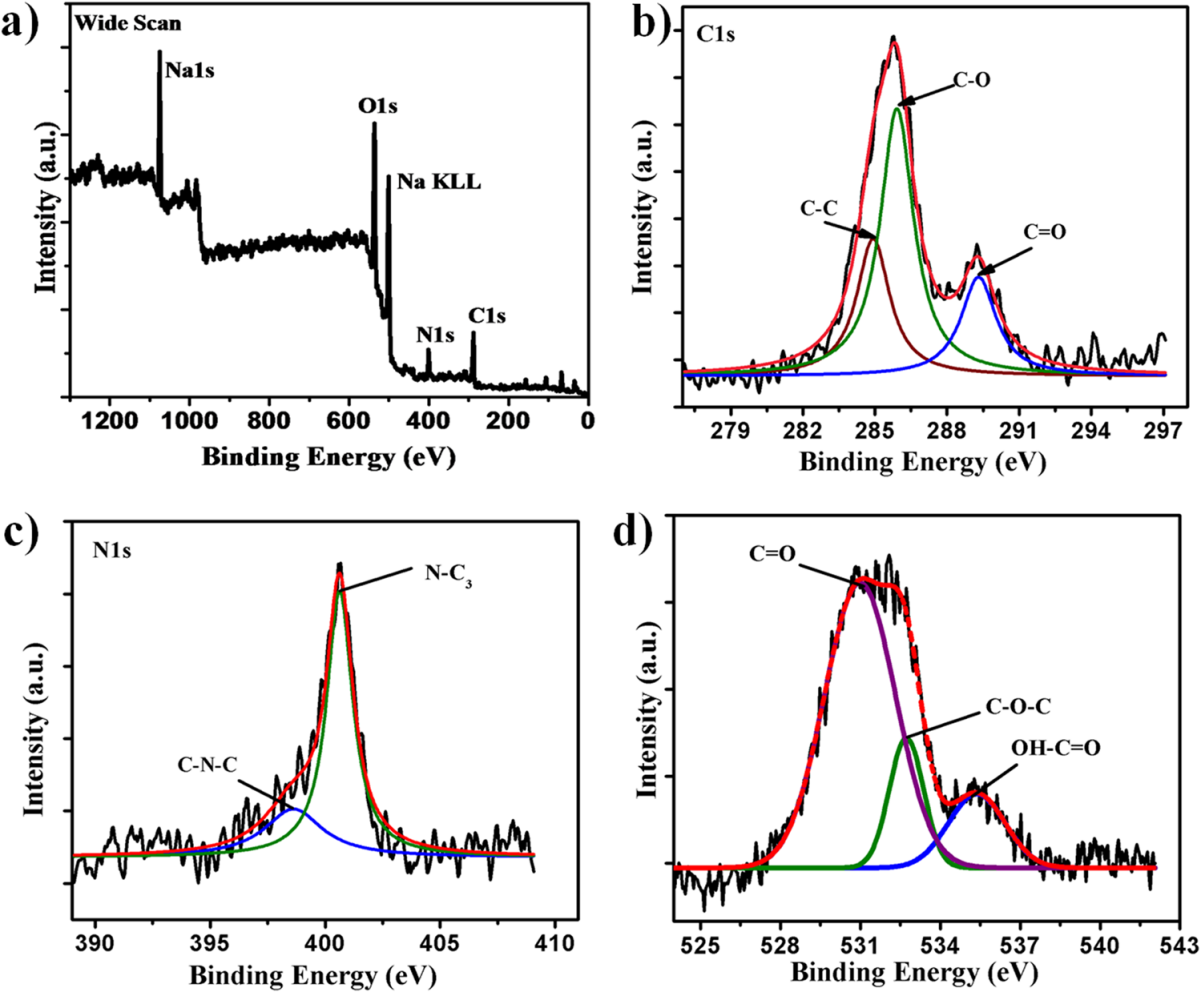
XPS analysis of CDs (**a**) Survey scan; High resolution spectrum of (**b**) Carbon C1s, (**c**) Nitrogen N1s and (**d**) Oxygen O1s.

The chemical composition and surface states of synthesized CDs was clarified by XPS spectrum. As shown in XPS scan (Fig. 2a), there are three characteristic peaks at 285.4, 400.01 and 532.3 eV which are attributed to C1s, N1s and O1s respectively. The content of carbon, nitrogen and oxygen at their respective peak positions are 74.91, 5.32, and 20.77% respectively. However, two more peaks observed in XPS spectrum are assigned for the sodium element and are adventitious since it is used for neutralisation of CDs solution. The C1s spectrum (Fig. 2b) was deconvoluted into three peaks at 284.8, 285.9, and 289.3 eV, and are due to the C=C/C–C, C–O and C=O bonds respectively. Remarkably, 284.8 eV energy peak is owing to the either sp^2^ or sp^3^ carbon atoms indicated synthesized CDs possessed predominantly both carbons. High-resolution XPS spectra of N1s spectrum (Fig. 2c) displayed two binding energy peaks at 398.7 and 400.6 eV can be attributed to C–N–C and N–C/N–H groups respectively on the surface of CDs^26^. Deconvoluted high resolution spectra O1s (Fig. 2d) exhibited three main binding energy peaks at 530.9, 532.7 and 535.4 eV confirming C=O, C–OH/C–O–C=C–OH respectively^27^. These obtained XPS results are consistent with FTIR spectroscopic analysis which suggested that the synthesized CDs have numerous hydroxyl and carboxyl functionality.

### Optical properties of CDs

**Figure 3:**
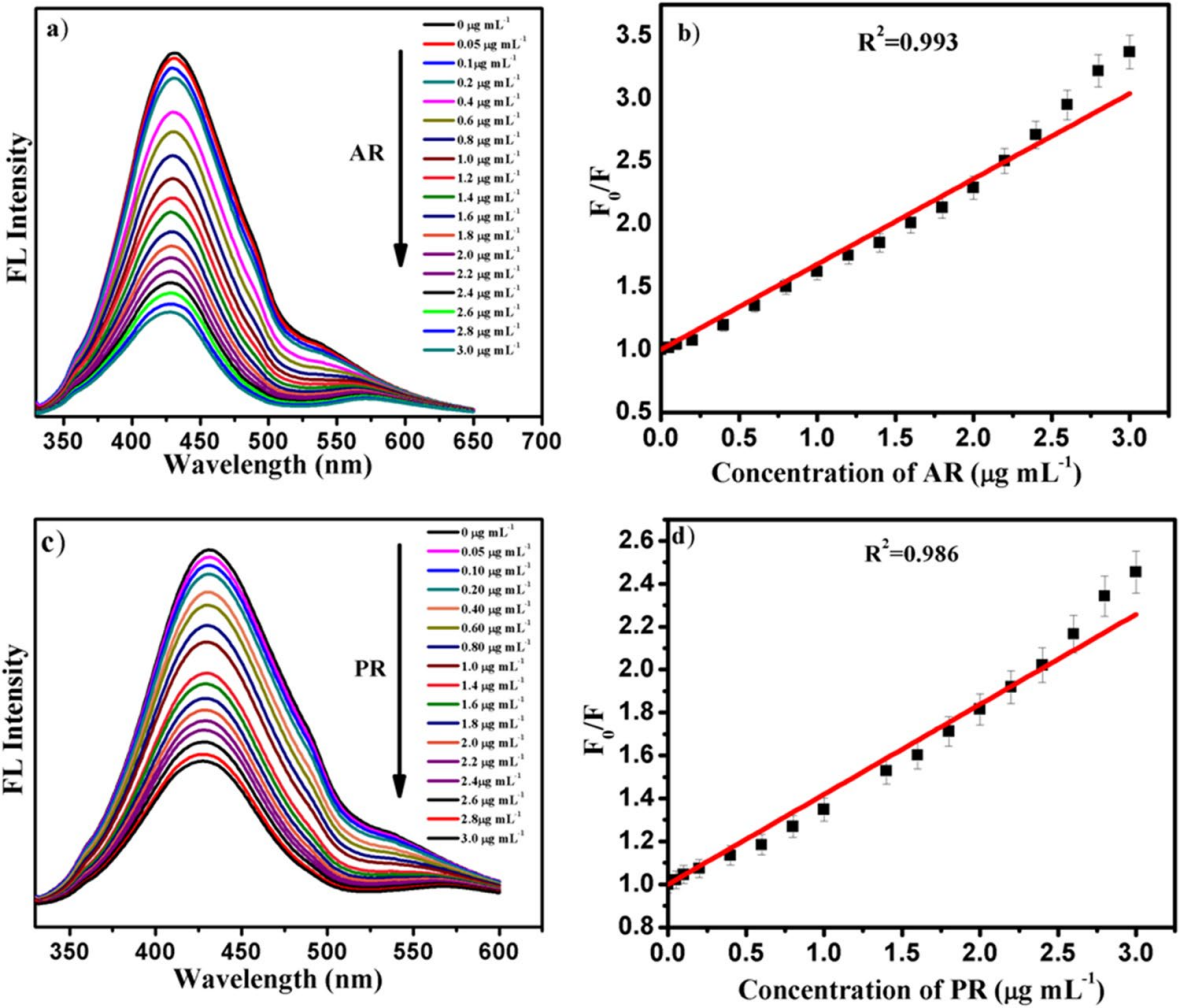
Fluorescence quenching phenomenon of CDS with different concentrations of (**a**) allura red (**c**) ponceau -4R dyes; (**b**, **d**) are their respective calibration curves.

The optical properties of CDs were characterised by absorption and fluorescence emission spectra. FigureS3a displays UV–Vis absorption and fluorescence spectra of CDs. Absorption spectrum shows shoulder like a peak at around 300 nm, attributed to the n–π* transition of surface C=O group of CDs^28^. Meanwhile, upon excitation at 320 nm, CDs exhibited excellent fluorescence emission at 430 nm. Therefore, fluorescence property of CDs was studied by varying the excitation wavelength within 290–410 nm (Figure S2b). This spectrum shows red shift in emission wavelength with excitation. That means, synthesised CDs exhibit excitation dependent fluorescence which is characteristic feature of CDs. This phenomenon is widely observed in fluorescent carbon nanomaterials and attributed to the different surface defect states near the Fermi level of CDs^29^. Additionally, fluorescence lifetime of prepared CDs is recorded and it is observed 1.67 ns (Figure S3c). All these optical properties are characteristic of CDs and are well matched with reported articles.

### Stability of CDs

The luminescent property of the CDs in different pH solutions was explored. Synthesised CDs displays almost stable photoluminescence within 4–12pH range while at strongly acidic (2–3 pH) condition shows noticeable effect on fluorescence intensity [Figure S4a]. This pH-dependent photoluminescent behaviour is attributed to the electron donors and electron acceptors behaviours of CDs^25^.The ionic strength of solution is a crucial factor so their effect on the emission intensity of CDs was investigated at different concentrations of NaCl (0–1.0 M). Figure S4b shows no obvious change of emission intensity of CDs under these ionic solutions. Moreover, continuous excitation at 320 nm for 60 min do not have profound effect on the photoluminescence of CDs (Figure S4c). In addition, long-term storage stability of CDs is also explored. As shown in figure S4d the emission intensity was almost unabated throughout storage time (up to 70 days) indicates a long-term homogeneous phase without any aggregation of CDs. All these results elucidate that synthesised CDs possess remarkably stability to the highly ionic solutions, shows good tolerance for pH, photostable and no effect of storage time, and eventually finds the numerous applications in various fields.

### CDs as probe for AR and PR sensing

Abundant availability of hydroxyl and carboxyl functional groups on CDs and owing to their interaction with analytes finds many applications in numerous fields. Sensing is one of them which was evaluated by monitoring the initial fluorescence intensity of CDs and eventually for the detection of different analytes. It has been identified that the high selectivity of CDs for AR and PR synthetic colorants is due to the complex formation through hydrogen bonding between them^30^. As shown in Fig. 3a,c, intensity of CDs decreases gradually with the increase in the concentration of AR and PR. The emission intensity was notably quenched (up to 75 and 80%) by both the 3.0 µg mL^−1^ colorants. This quenching phenomenon was further demonstrated by Stern Volmer type equation. An excellent linear relationship between concentrations of dyes and quenching factors F_0_/F in the range of 0.0 to 3.0 µg mL^−1^ was observed with following equations (Fig. 3b,d).

$$ {\text{F}}/{\text{F}}0 = {\text{ }}0.679{\text{ CAR }} + 1{\text{ }}(R^{2}  = 0.993);\;{\text{F}}/{\text{F}}0 = {\text{ }}0.419{\text{ CPR }} + 1{\text{ }}(R^{{\text{2}}}  = 0.986) $$

where F_0_ and F are the fluorescence intensity of CDs at 430 nm in the absence and presence of dyes. The limit of detection (LOD) for dyes were estimated by 3σ/k and was found to be 0.45 and 0.47 μg mL^-1^ for AR and PR colorants respectively. The wide linear range and lowest detection limit of the developed method was enough to monitor AR and PR synthetic dyes in different food products since maximal acceptable concentrations of both the synthetic colorants lies in the working range. (European Food Safety Authority; AR- and PR-), thus this fluorescent probe could be employed for sensitive quantification of dyes in food products. We also compared our method for the quantification of AR and PR synthetic dyes with existing method and found that developed new approach is in good agreement with them (Table S1).

**Figure 4:**
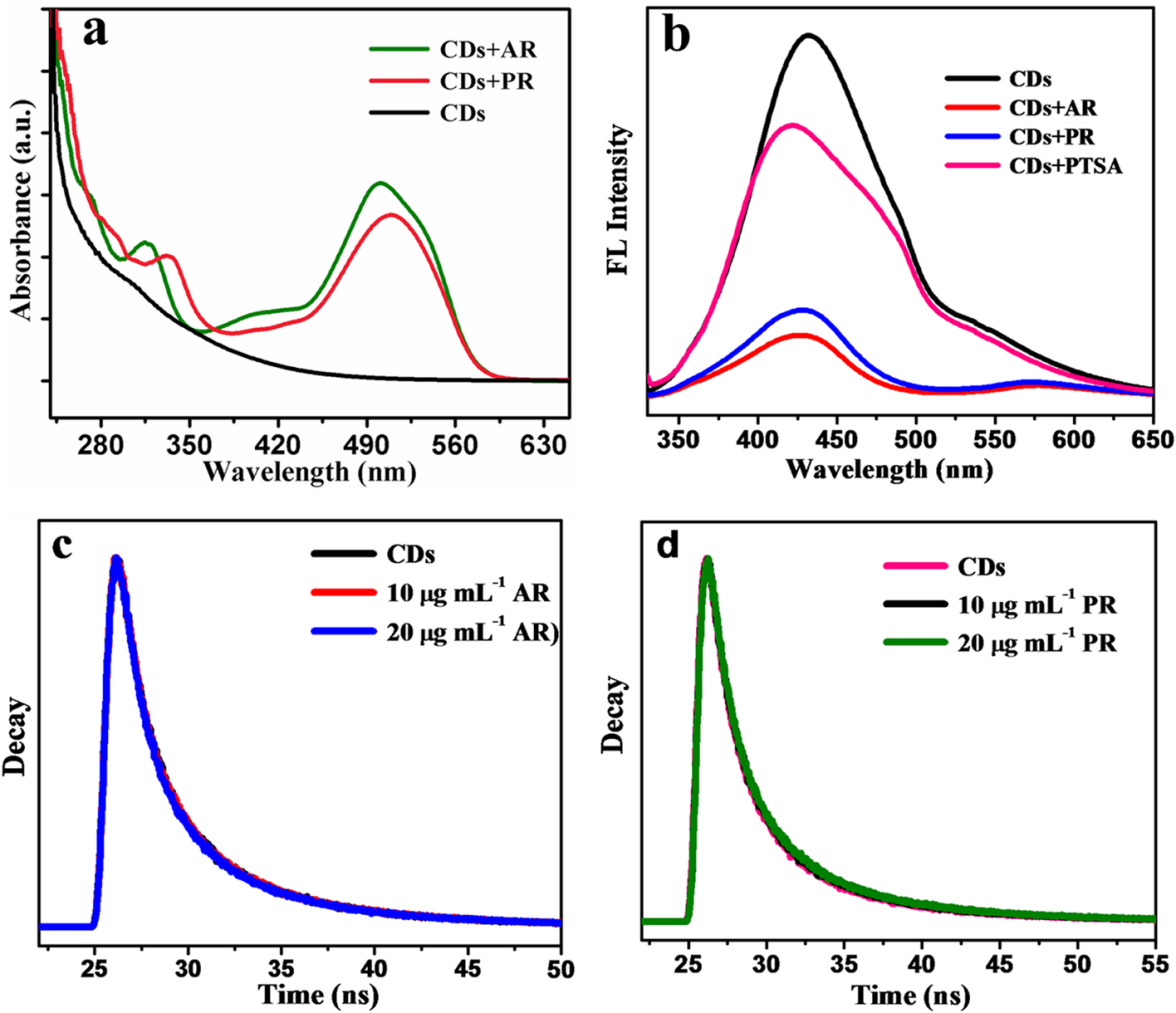
Interpretation of mechanism of quenching through; (**a**) Absorption spectrum (**b**) fluorescence spectrum; life time spectrum with AR dye (**c**) and with PR dyes (**d**).

The insights of fluorescence quenching mechanism were evaluated by considering surface functional groups of CDs and both dyes. The carboxyl and or hydroxyl group of CDs and sulfonic group or hydroxyl group of analyte may forms a complex between them through the hydrogen bonding. Thus, this complexation is the main reason for quenching of fluorescence of CDs^26^. This mechanism was further illustrated by optical characteristics of both probe and analyte. The shoulder like peak of CDs at 300 nm in absorption spectrum (Fig. 4a) was diminished with addition of dyes, suggested the formation of complex between them. Moreover, fluorescence spectra of CDs showed blue shift in presences of both the synthetic dyes also confirms the complexation. In addition, to address the complex formation, we have used another sulfonic group containing para toluene sulfonic acid (PTSA) compound. From Fig. 4b, it showed quenching of fluorescence of CDs with blue shift in presences of PTSA, affirmed that interaction between sulfonic group and carboxyl group may be the prime cause of fluorescence quenching. Fluorescence quenching could be either ground state complex (static type) or excited state complex (dynamic type) formation. The discrimination between these two modes of quenching was identified by fluorescence decay experiment. Figure 4c,d depicts decay profile of the CDs in the presence of different concentrations of AR and PR dyes respectively. These figures showed almost no change in average fluorescence lifetime of CDs with different concentrations of dyes, indicates static type of quenching process.

### Effect of foreign substances

**Figure 5:**
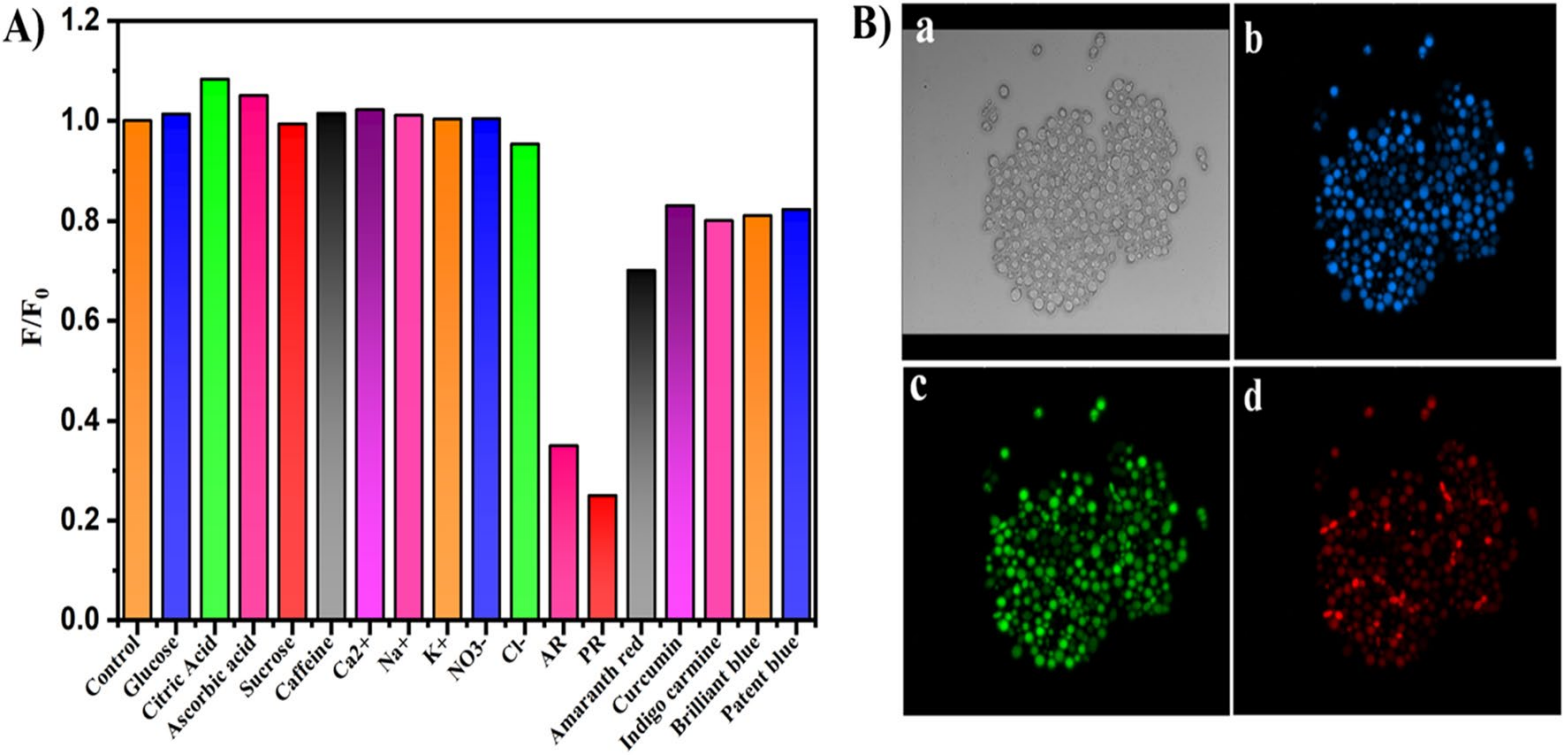
(**A**) Study of effect of interfering ions and molecules on fluorescence quenching of CDs (**B**) Confocal laser microscopic images of S. cerevisiae treated with CDs in bright field (**a**), and at excitation wavelengths (**b**) 408 (blue), (**c**) 488 (green), and (**d**) 561 (red) nm.

Food additives such as sweeteners and preservatives are the prime source of interferences in analysis of food samples. Therefore, selectivity of the proposed method was demonstrated with these possible interfering substances on determination of AR and PR dyes. Figure 5A shows the response of CDs to the both the colorants were not affected by different molecules such as glucose, citric acid, ascorbic acid, sucrose, caffeine and ions such as Ca^2+^, Na^+^, K^+^, NO_3_^−^ and Cl^-^ with relatively higher concentration and some dyes such as amaranth red, curcumin, indigo carmine, patent blue, brilliant blue etc. These results suggest that, proposed method exhibit excellent selectivity towards the dyes and hence could be employed to quantify the AR and PR in real samples.

### Analytical application

Success of every developed method lies in their applicability. Therefore, the performance of the developed method was evaluated in two different soft drink samples by the standard addition method. The analysis results are listed in Table 1. The recoveries of AR and PR ranged from 98.7–101.2%and 99–101% respectively with comparable relative error. Moreover, the relative standard deviation (RSD) was found to be lower than 3% demonstrated that the excellent reproducibility of this method. These results such as good accuracy and precision suggest that analytical performance for quantification of AR and PR are satisfactory in soft drink samples and eventually proved very convenient method in real samples analysis of AR and PR synthetic dyes.

**Table 1:** Determination of Dyes in soft drinks by Standard addition method.

Samples	Dyes	Amount of Sample (mL)	Amount of standard dyes (µg mL^-1^)	Total dyes Found (µg mL^-1^) (n = 3)	Recovery of Pure ETA (%) (n = 3)	RSD (%)	Relative Error (%)
Soft drink No.1	AR	1.0	1.0	0.99	99.57	0.82	− 0.004
		1.0	1.5	1.51	100.54	1.14	0.005
		1.0	2.0	1.94	97.09	2.79	1.27
	PR	1.0	1.0	0.98	98.84	2.20	− 0.011
		1.0	1.5	1.48	98.86	2.55	− 0.011
		1.0	2.0	1.98	99.22	1.37	− 0.155
Soft drink No.2	AR	1.0	1.0	0.97	98.82	0.58	− 0.011
		1.0	1.5	1.49	99.05	1.72	− 0.009
		1.0	2.0	1.95	97.89	0.751	− 0.022
	PR	1.0	1.0	1.01	100.14	2.53	0.001
		1.0	1.5	1.51	100.86	1.79	0.008
		1.0	2.0	1.97	99.37	1.24	− 0.006

### Live cell imaging

The cells of microorganism can be effectively and vividly visualised under fluorescence microscopy if it is stained with fluorescent material. In this context, CDs have become interesting imaging material because of their physical, intrinsic fluorescence and tuneable emission properties. Therefore in an attempt, sawmill waste derived CDs have been explored as imaging probe for S. cerevisiae cells. Figure 5B displays confocal microscopic images of yeast cells (S. cerevisiae), incubated with CDs (40 μL) for 3 h, under bright field and different excitation wavelengths. The images exhibited strong blue, green and red emission [Fig. 5B (b,c,d)] when excited at 408, 488, and 561 nm wavelength respectively. The highly fluorescent nature of these images under microscopy are due to the penetration of CDs through the cell membrane by endocytosis mechanism and eventually homogeneous distribution in the cell cytoplasm. This result illustrate that CDs has the ability to stain the yeast cells and can be acts as potential candidate for multicolour imaging and biological labelling of S. cerevisiae.

## Conclusion

In conclusion, abundantly available saw mill waste has been used for the synthesis of CDs by chemical oxidation method. The as synthesised CDs have exhibited excellent water solubility, comparable quantum yield and stability in ionic medium and photostability against the irradiating light. Furthermore, CDs is employed as a fluorescent probe for the quantification of AR and PR dyes. The calibration curve indicated wide range linearity and considerable LOD for the detection of both synthetic colorants. Because of this, the developed method was applied for the detection of dyes in real soft drink samples. The good accuracy and precision suggested the suitability of the method for real sample analysis. In addition this, CDs also employed for cell imaging study. The observed data suggested that CDs acts as an excellent material for multicolour staining of cells.

## Supplementary Information

Supplementary Information.

## References

[1] Dinç E, Baydan E, Kanbur M, Onur F (2002). Spectrophotometric multicomponent determination of sunset yellow, tartrazine and allura red in soft drink powder by double divisor-ratio spectra derivative, inverse least-squares and principal component regression methods. Talanta.

[2] Kemp A (2008). BMJ.

[3] Shimada C (2010). Differential colon DNA damage induced by azo food additives between rats and mice. J. Toxicol. Sci..

[4] Sorouraddin MH, Rostami A, Saadati M (2011). A simple and portable multi-colour light emitting diode based photocolourimeter for the analysis of mixtures of five common food dyes. Food Chem..

[5] Liao QG (2012). Applicability of accelerated solvent extraction for synthetic colorants analysis in meat products with ultrahigh performance liquid chromatography–photodiode array detection. Anal. Chim. Acta.

[6] Wu H (2013). A rapid shaking-based ionic liquid dispersive liquid phase microextraction for the simultaneous determination of six synthetic food colourants in soft drinks, sugar- and gelatin-based confectionery by high-performance liquid chromatography. Food Chem..

[7] Chanlon S (2005). Determination of Carmoisine, Allura red and Ponceau 4R in sweets and soft drinks by differential pulse polarography. J. Food Compos. Anal..

[8] Li K (2017). New LC-MS/MS Method for the analysis of Allura Red level in takeaway chinesedishes and urine of an adult chinese population. J. Agri. Food Chem..

[9] Zhang Y (2010). Multi-wall carbon nanotube film-based electrochemical sensor for rapid detection of Ponceau 4R and Allura Red. Food Chem..

[10] Zhang J (2015). Simultaneous determination of Ponceau-4R and Allura Red in soft drinks based on the ionic liquid modified expanded graphite paste electrode. Int. J. Environ. An. Ch..

[11] Guo Y (2015). Fluorescent carbon nanoparticles for the fluorescent detection of metal ions. Biosen. Bioelectron..

[12] Xu JJ (2014). Functional nanoprobes for ultrasensitive detection of biomolecules: an update. Chem. Soc. Rev..

[13] Zuo P (2016). A review on syntheses, properties, characterization and bioanalytical applications of fluorescent carbon dots. Microchim. Acta..

[14] Esteves JCG, Gonçalves HMR (2011). Analytical and bioanalytical applications of carbon dots. Trend. Anal. Chem..

[15] Sun X, Lei Y (2017). Fluorescent carbon dots and their sensing applications. Trend. Anal. Chem..

[16] Wang R (2017). Recent progress in carbon quantum dots: synthesis, properties and applications in photocatalysis. J. Mat. Chem. A..

[17] Zhang J, Yu SH (2016). Carbon dots: Large-scale synthesis, sensing and bioimaging. Mater. Today.

[18] Zeng Q (2016). Carbon dots as a trackable drug delivery carrier for localized cancer therapy in vivo. J. Mater. Chem. B..

[19] Siyu L (2017). Near-infrared photoluminescent polymer-carbon nanodots with two-photon fluorescence. Adv. Mater..

[20] Li W (2019). Kilogram-scale synthesis of carbon quantum dots for hydrogen evolution, sensing and bioimaging. Chin. Chem. Lett..

[21] Wang B (2019). Near-infrared emissive carbon dots with 3396% emission in aqueous solution for cellular sensing and light-emitting diodes. Sci. Bull..

[22] Song H (2019). High production-yield solid-state carbon dots with tunable photoluminescence for white/multi-color light-emitting diodes. Sci. Bull..

[23] Qiao ZA (2010). Commercially activated carbon as the source for producing multicolor photoluminescent carbon dots by chemical oxidation. Chem. Comm..

[24] Zhao S (2017). Green synthesis of bifunctional fluorescent carbon dots from garlic for cellular imaging and free radical scavenging. ACS Appl. Mater. Interfaces..

[25] Jones S, Sahatiya P, Badhulika S (2017). One step, high yield synthesis of amphiphilic carbon quantum dots derived from chia seeds: a solvatochromic study. New J. Chem..

[26] Atchudan R (2017). Facile green synthesis of nitrogen-doped carbon dots using Chionanthus retusus fruit extract and investigation of their suitability for metal ion sensing and biological applications. Sens. Actuat. B Chem..

[27] Lan M (2015). A recyclable carbon nanoparticle-based fluorescent probe for highly selective and sensitive detection of mercapto biomolecules. J. Mater. Chem. B..

[28] Nie H (2014). Carbon dots with continuously tunable full-color emission and their application in ratiometric pH sensing. Chem. Mater..

[29] Wang N (2016). Green preparation of carbon dots with papaya as carbon source for effective fluorescent sensing of Iron(III) and Escherichia coli. Biosens. Bioelectron..

[30] Zhang J (2016). Graphene quantum dots as a fluorescence-quenching probe for quantitative analysis of Ponceau 4R solution. Anal. Methods.

