# Supplementary material for: Sawmill waste derived carbon dots as a fluorescent probe for synthetic dyes in soft drinks
Source: Sci Rep. 2021 Sep 9;11:17996. doi: 10.1038/s41598-021-97552-5 (PMC8429643; doi:10.1038/s41598-021-97552-5)
Supplement: Supplementary file 1 — Supplementary Information. [file 41598_2021_97552_MOESM1_ESM.docx]

**Supplementary Information**

**Sawmill Waste Derived Carbon Dots as a Fluorescent Probe for Synthetic Dyes in Soft Drinks**

Datta B. Gunjal^1,2^, Laxman S. Walekar^1^, Samadhan P. Pawar^1^, Prashant V. Anbhule^1^, Mukund G. Mali^3^, Vinayak P. Dhulap^4^, Daewon Sohn^5^, Prasad G. Mahajan^6^, Ki Hwan Lee^7^, Rajendra V. Shejwal^*2^ and Govind B. Kolekar^*1^

^1^*Fluorescence Spectroscopy Research Laboratory, Department of Chemistry, Shivaji University, Kolhapur-416 004, Maharashtra, India*

^2^*Department of Chemistry, Lal Bahadur Shastri College of Arts, Science and Commerce, Satara-415002, Maharashtra, India.*

^3^*School of Chemical Sciences, Punyashlok Ahilyadevi Holkar, Solapur University, Solapur-413255, Maharashtra, India.*

*^4^School of Earth Sciences, Punyashlok Ahilyadevi Holkar, Solapur University, Solapur-413255, Maharashtra, India*

*^5^Department of Chemistry and the Research Institute for Convergence of Basic Science, Hanyang University, Seoul, 04763 Republic of Korea.*

*^6^Vidya Prathisthan’s Arts, Commerce & Science College, Vidyanagari, Baramati-413133, Maharashtra, India.*

*^7^Department of Chemistry, Kongju National University, Gongju, Chungnam 32588, Republic of Korea.*

**Figure. S1** Schematic presentation of Synthesis of CDs and their different applications


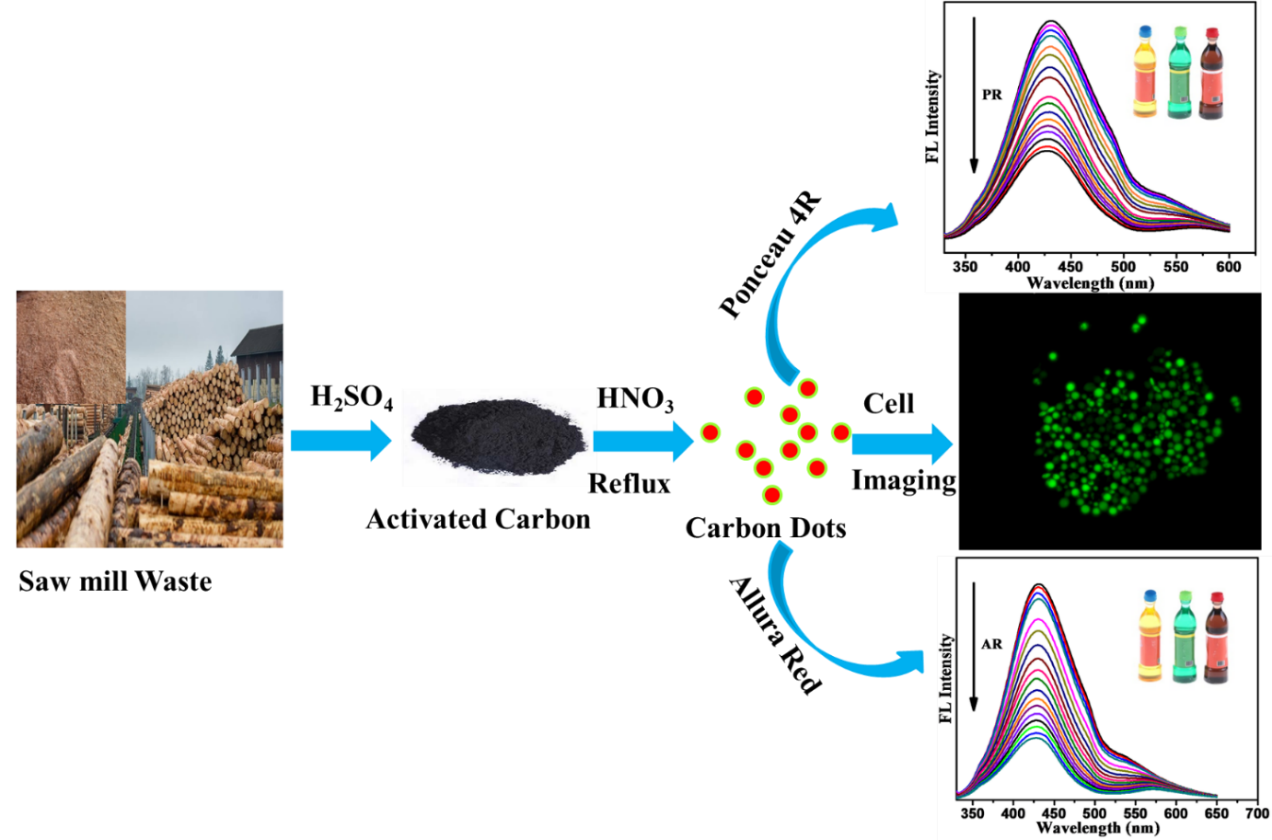


**Figure S2.** Dynamic Light Scattering measurement of CDs

**
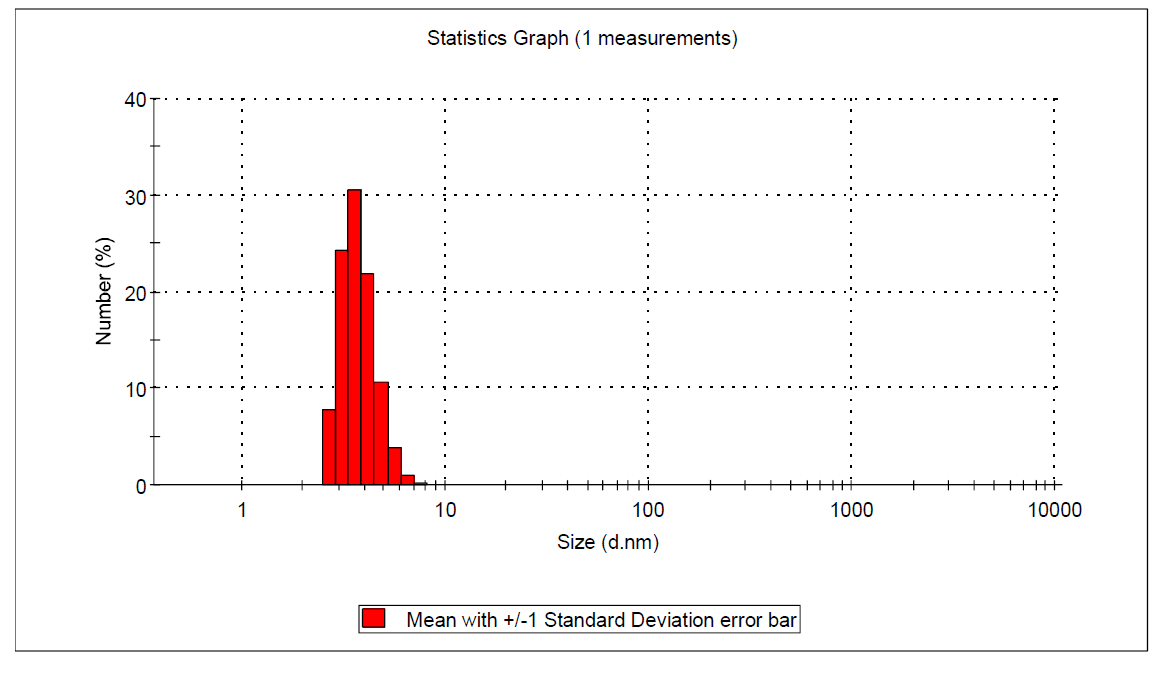
**

**Figure S3.** Optical spectra of CDs a) Uv-Vis absorption, Excitation and Emission Spectrum; b) Excitation dependent emission behaviour of prepared CD;

c) Fluorescence decay profile of CDs


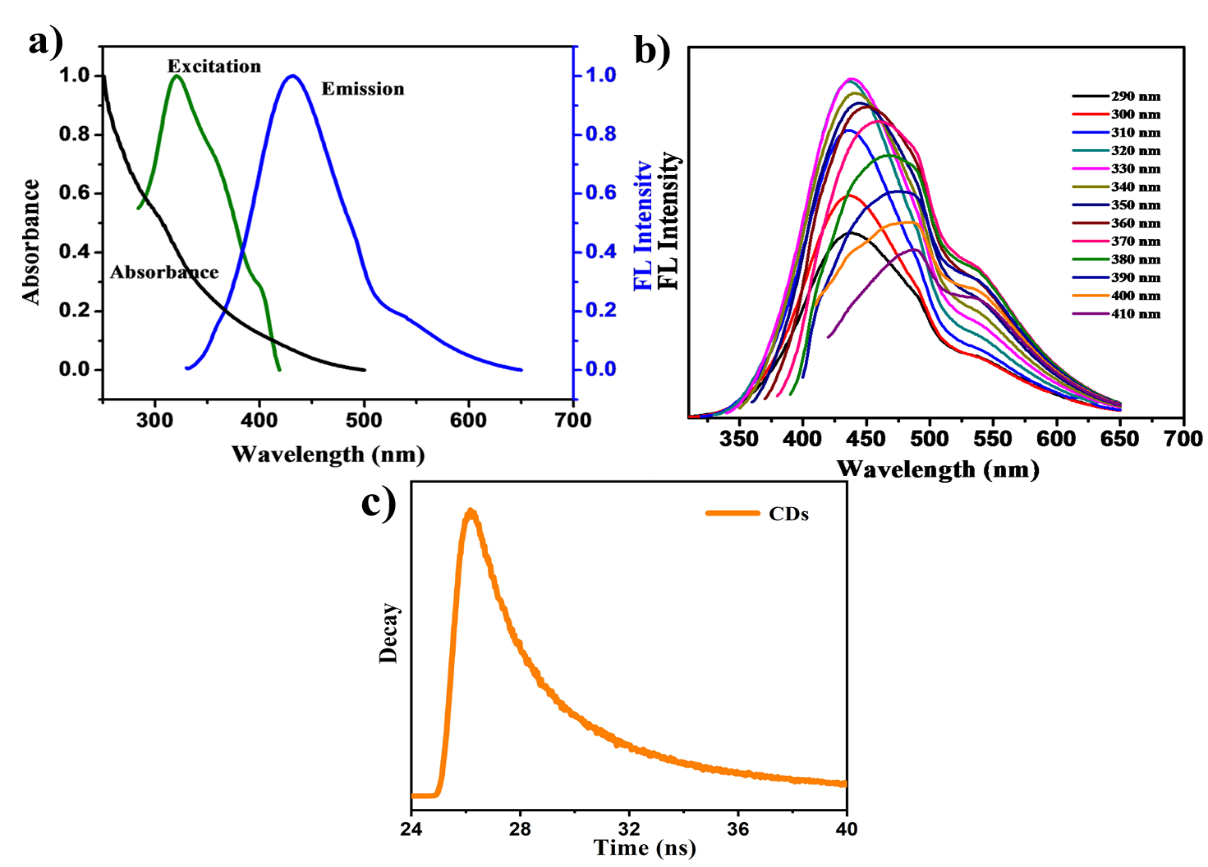


**Figure S4.** Stability Study a) Effect of pH b) Effect of ionic strength c) Effect of Irradiation Time and d) Effect of Storage time on fluorescence intensity of CDs.


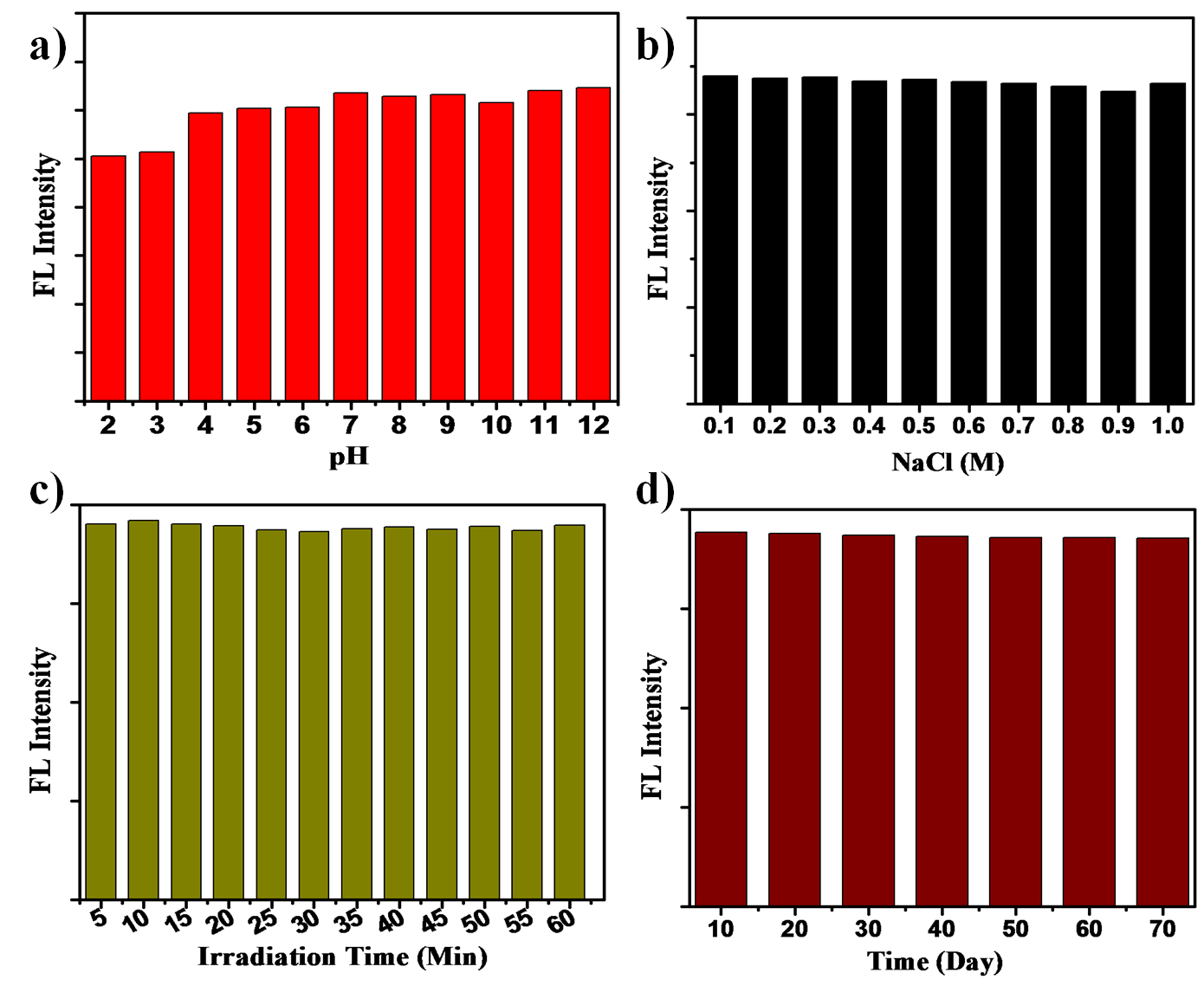


**Table S1.** Comparison of different methods for the detection of Allura red and Ponceau 4R

| **Material/ Method** | **Limit of Detection** | **Linear Range** | **Dyes** | **Applications** | **References** |
| --- | --- | --- | --- | --- | --- |
| Carbon Dots | 0.45 and 0.47 µg mL^-1^ | 0.0 to 3.0µg mL^-1^ | Allura red and Ponceau 4R | Soft Drinks | **This Method** |
| Graphene quantum dots | 2.57 μg mL^-1^ | 5-150 μg  mL^-1^ | Ponceau 4R | Beverage and Water | Zhang, J. *et al.* |
| MWCNT | 15 and 25 µg L^-1^ | 25 to 1500  µg L^-1^and  50 to 600  µg L^-1^ | Ponceau 4R and Allura red | Soft Drinks | Zhang, Y. *et al.* |
| Ɛ-MnO2 microspheres/chitosan /GCE | - 1. nmol   dm^-3^ | 0.005 to 1000 mmol dm^-3^ | Ponceau 4R | Soft Drinks | Huang, J. *et al.* |
| Gold Nanorods | 0.10 mg  L^-1^ | - 1. – 5.00   mgL^-1^ | Allura red | Beverage | Ou,Y. *et al.* |
| Graphene andnickel nanoparticles modified electrode | 8.0 nmol  L^-1^ | 0.05–10.0  mol L^-1^ | Allura red | Strawberry juice | Yu, L. *et al.* |
| HPLC | 32µg L^-1^ | 50–20,000  µg L^-1^ | Allura red | Drinksand Candies | Yoshioka, N. *et al.* |

**References:**

1. Zhang, J. *et al.* Graphene quantum dots as a fluorescence-quenching probe for quantitative analysis of Ponceau 4R solution. *Anal. Methods* **8,** 7242-7246 (2016).
2. Zhang, Y. *et al.* Multi-wall carbon nanotube film-based electrochemical sensor for rapid detection of Ponceau 4R and Allura Red. *Food Chem.* **122,** 909-913 (2010).
3. Huang, J., Zeng, Q. & Wang, L. Ultrasensitive electrochemical determination of Ponceau 4R with a novel e-MnO2 microspheres/chitosan modified glassy carbon electrode. *Electrochim. Acta.* **206,** 176-183 (2016).
4. Ou, Y. *et al.* Gold nanorods as Surface-Enhanced Raman Spectroscopy (SERS) substrates for rapid and sensitive analysis of allura red and sunset yellow in beverages. *J. Agri. Food Chem.* **66,** 2954-2961 (2018).
5. Yu, L. *et al.* Detection of allura red based on the composite of poly(diallyldimethylammoniumchloride) functionalized graphene andnickel nanoparticles modified electrode, *Sens. Actuators B: Chem.* **225,** 398–404 (2016).
6. Yoshioka, N. & Ichihashi, K. Determination of 40 synthetic food colors in drinks and candies by high-performance liquid chromatography using a shortcolumn with photodiode array detection. *Talanta* **74,** 1408-1413 (2008).
